# Potential confounders in linking elevated S100A8/A9 to left ventricular dysfunction in septic shock patients

**DOI:** 10.1186/s13054-023-04769-z

**Published:** 2023-12-06

**Authors:** Patrick M. Honore, Emily Perriens, Sydney Blackman

**Affiliations:** 1 CHU UCL Godinne Namur, UCL Louvain Medical School, Campus Godinne, Avenue G Thérasse 1, 5530 Yvoir, Belgium; 2 ULB University, Brussels, Belgium

Jakobsson et al. investigated the role of S100A8/A9, a pro-inflammatory alarmin, in sepsis-induced myocardial dysfunction (SIMD). They concluded that elevated S100A8/A9 is associated with the development of left ventricular (LV) dysfunction in severe sepsis patients [1].

Patients 18 years of age and older admitted to the intensive care unit (ICU) with septic shock (per Sepsis III) were included in this study [1]. Thirty-five out of sixty-two (56%) patients had LV dysfunction. Plasma S100A8/A9 was significantly higher in LV dysfunction patients (20.1 μg/mL vs. 7.4 μg/mL, *P* = 0.009). Nearly half of critically ill patients, especially with septic shock, develop acute kidney injury (AKI), and 20–25% require renal replacement therapy (RRT) within the first ICU week [2]. Considering S100A8 (10.8 kDa) and S100A9 (13.2 kDa) molecular weights, as well as the molecular weight of the S100A8/A9 heterodimer (24 kDa) [3], continuous RRT (CRRT)—which has a cut-off value of 35–40 kDa—might eliminate these molecules, impacting bio marker levels, and potentially leading to artificially decreased S100A8/A9 levels [4, 5]. The absence of CRRT/RRT in the criteria and its impact on each group is a potential major comfounding factor that could heavily influence results [4, 5]. In a clinical setting, this could lead to inaccurate prognosis and unadapted support. It is necessary that a sensitivity analysis should be done after the exclusion of CRRT/RRT patients to clarify the performance of these biomarkers when they are not artificially removed by an extracorporeal purification technique [5].

## Data Availability

Not applicable.

## Abbreviations

LV: Left ventricular
ICU: Intensive care unit
AKI: Acute kidney injury
RRT: Renal replacement therapy
CRRT: Continuous renal replacement therapy
